# Franz Joseph Gall on the Cerebellum as the Organ for the Reproductive Drive

**DOI:** 10.3389/fnana.2019.00040

**Published:** 2019-04-16

**Authors:** Paul Eling, Stanley Finger

**Affiliations:** ^1^Donders Institute for Brain, Cognition and Behaviour, Radboud University Nijmegen, Nijmegen, Netherlands; ^2^Department of Psychological and Brain Sciences, Program in History of Medicine, Washington University, Saint Louis, MO, United States

**Keywords:** Gall (Franz Joseph), cranioscopy, mental faculties, localization of function, cerebellum, reproduction, Flourens (Marie Jean Pierre), movement disorders

## Abstract

Franz Joseph Gall (1758–1828) is best remembered for his belief that bumps on the skull reflect the growth of small, underlying brain areas, though among some historians, more positively for introducing the concept of cortical localization of function. All but one of Gall’s 27 settled-upon cortical faculties involved the cerebral cortex, the exception being his most primitive faculty, reproductive instinct, which he associated with the cerebellar cortex. This article examines Gall’s earlier subcortical organs, with an emphasis on why he associated the cerebellum with this drive. It draws from accounts by several physicians, who attended his Vienna lectures or heard him speak in Germany and the Netherlands in 1805–1806 [i.e., before he published his finalized list in his *Anatomie et Physiologie* (1810–1819)]. These early accounts show that early on he localized at least four faculties in brainstem structures, including a reproductive drive in the cerebellar cortex. He based his structure–function association primarily on cranial differences between men and women, and what he found in males and females of other species, although cranioscopy was not his sole method. It is also shown that, in opposition to his cerebellar–reproductive drive association, Marie Jean Pierre Flourens linked coordinated skeletal movements to the cerebellum after conducting lesion experiments, mainly on birds. Flourens did not design his experiments to challenge Gall’s ideas on localization of function, but they did just that. Gall responded that ablation methods lack precision and lead to misguided conclusions. How Gall continued to associate the reproductive instinct with the cerebellar cortex, even after deleting his other brainstem-based associations from his faculties of mind, tells us much about him and the faith he had in his methods and doctrine.

## Introduction

**Figure 1:**
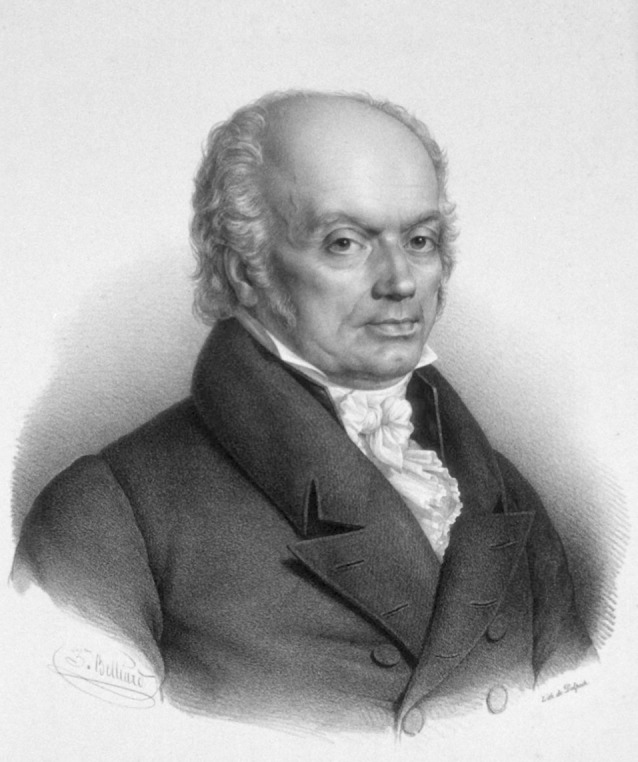
Franz Joseph Gall (1758–1728; picture from the public domain).

Franz Joseph Gall (1758–1828; Figure 1) was born in Tiefenbronn, a small German town, and although it was expected that he would enter the priesthood, he opted to pursue medicine in Strasbourg and then Vienna (for Gall’s biography, see Finger and Eling, 2019). After he completed his medical degree in 1785, he immediately started a private practice in a fashionable part of Vienna. But more than just wanting to treat patients, he aspired to make a name for himself in science and medicine, and, more specifically, to come forth with a new, empirical science of humankind. Hence, he used some of his earnings to support a rapidly expanding research program aimed at understanding the mind, the brain, and group and individual differences in behavior.

In 1791, Gall published the first volume of a small book showing the need for an empirical approach to medicine (Gall, 1791). In it, he dispensed of metaphysics and briefly alluded to the possibility of there being more than just a few independent faculties of mind, such as the time-honored concepts of perception, cognition, and memory. But he did not name any alternative faculties, and he wrote nothing about craniology, which would soon emerge as his primary method for correlating brain structures with specific functions.

Five years later, and still without an academic appointment, he began to lecture about the doctrine now firmly tied to his name, doing so at his stately home, where he had already begun to build a sizeable collection of skulls and head casts, along with some brain casts. Warmly received by those that listened to what he had to say, he proceeded to lay out the fundamentals of his revolutionary doctrine in a published letter to the Viennese censor (who endorsed what he was doing) in 1798. In it, he discussed practical (as opposed to abstract) faculties of mind, localization of function, and how his theory was based on extreme cases (e.g., criminals, the insane, gifted musicians, etc.)—while expressing a need for more skulls and casts from famous people. At the same time, he brought up the importance of studying animal behaviors and skulls in his far-reaching, nature-based research program.

Everything seemed to be going well for Gall until the end of 1801 when the Holy Roman Emperor attempted to put an end to his lecturing. He was charged with two things: lecturing without permission and teaching dangerous materialism. He maintained these were absurd charges, but to his dismay his detailed written rebuttal was dismissed, hampering his ability to inform people of his insights and to get the feedback he needed for a book he seemed to have started on his doctrine.

Realizing what he was up against, Gall left Vienna in 1805 and began a lecture-demonstration tour through what is now Germany, Denmark, the Netherlands, and Switzerland. In many places, he drew large paying crowds, and he was also showered with other rewards (e.g., gifts of gold coins, watches, medals), including some by kings and queens. Gall seems to have expected to return to Vienna within a year, during which he would also have the opportunity to see his parents, but he never returned to Austria, even when invited back later in life.

In 1807, Gall entered Paris, where he would later become a French citizen and now reside until his death in 1828. In fact, he made only one short visit outside of France during this time, and it was in 1823 to lecture in London.

A year after arriving in Paris, he and Johann Gaspard Spurzheim (1776–1832), who had been his assistant since 1800, presented some of their anatomical discoveries about the brain to the *Académie des Sciences*, expecting a positive response from the organization officially charged with overseeing and policing the sciences in France. George Cuvier (1769–1832), the *Académie’s* secretary—ranking second only to its president, then Napoleon Bonaparte (1769–1821)—headed the committee evaluating the submission. Recognizing Napoleon’s stern warning that French scientists had no need of foreigners to teach them science or medicine (especially from countries they were then fighting), Cuvier took the position that there was not much new in Gall and Spurzheim’s submission (Finger and Eling, 2019). Incensed, Gall fought back, publishing a book of rebuttals in French and another in German, in which they included their submission, Cuvier’s response to it, and their rebuttal (Gall and Spurzheim, 1809a,b).

Also being criticized by Cuvier and others for not having laid out his doctrine in book form, as would be befitting a true scientist and not a charlatan, he and Spurzheim now focused on quickly completing their long-awaited book, which would cover both neuroanatomy and their more controversial “skull doctrine.” Their four-volume *Anatomie et Physiologie du Système Nerveux…* with its magnificent atlas containing 100 plates of brains and skulls of humans and lower animals, first started to appear in 1810, with the last volume being published 9 years later (Gall and Spurzheim, 1810–1819). During this time, Gall and Spurzheim parted ways, which is why both men are coauthors on the first two volumes and the atlas, and why Gall’s name appears alone on the third and fourth volumes.

Gall’s path to his mature doctrine (and his life story) has been told many times before, albeit briefly, though more recently in detail in a book by the current authors (Finger and Eling, 2019). Thus, we will not deal with the more general assumptions underlying his chosen faculties of mind, the cortical organs he associated with each higher function, and the like. Instead, we shall first focus on how his list evolved, starting early on with several brainstem organs, including the organ for reproductive drive in the cerebellum. Then we shall show how over time, he abandoned all of his subcortical organs except the one associated with the cerebellum, which became the first and most primitive organ of mind on his final list—and now the only one of his 27 organs not associated with the cerebral cortex.

## Gall’s Evolving List

The concept of multiple faculties of mind was not new in Gall’s day. Philosophers well before Gall characterized the soul or the mind in terms of its capacities or faculties, and so did his immediate predecessors and contemporaries. Some early philosophers even spoke and wrote about three types of souls (vegetative, animal, and rational), contending that only humans have the latter. This line of reasoning led to the widespread characterization of the rational soul as a force or agent responsible for perceiving, judging, and retaining information (memory), even among esteemed natural philosophers. The “hard problem” for such thinkers was and still is how to explain how an immaterial or ill-defined spiritual force could drive the physical machinery of the body (Smith and Whitaker, 2014).

Gall regarded perception, judgment, and memory as *abstract* faculties, further contending that people were basing the guiding soul and the very notion of life on their religious beliefs and on what had been passed down from authorities of the past. He rejected the classical interpretation of the broad capacities of the soul, along with all metaphysical conceptions, and instead offered his own set of practical faculties, which, in the best spirit of the Enlightenment, he considered testable and therefore scientific. Although he recognized perceiving and memory as important capacities, he argued that they are not primary or fundamental faculties. In contrast, he saw them as secondary features attached to other faculties. For instance, he viewed recognizing and producing music as a primary faculty, maintaining that perceiving music, making music, and retaining melodies are all functions of this faculty. The same could be said for mathematics, wit, religiosity, and other higher faculties.

By looking at the faculties of mind in this new, less abstract way, Gall felt he could explain why one person might excel in a singular domain (e.g., painting portraits, learning languages) and yet appear average or even deficient in other areas (e.g., mathematics, wit). It also allowed him to explain differences between species on the Great Chain of Being and among individuals within species. Understanding and explaining individual differences were, in fact, a major aim of his research program.

Gall’s thinking differed not only from traditional ideas about the mind but also from the empirical philosophy promoted by Englishman John Locke (1632–1704; Locke, 1690) and his followers. That line of thought placed more emphasis on learning (nurture) than on what could be ascribed to nature. Locke’s philosophy would lead to “sensationism” (or “sensationalism”) in France. Gall, however, would stress the importance of what he called “brain organization” and point to individual differences even in very young children—differences seemed to be passed down in families and remain stable over a lifetime.

In contrast to Locke, the French sensationalists, and the *Idéologues*, who followed them, all finding little reason to specify individual faculties, since everything was a matter of experience and learning, Gall began to come forth with a new list of primary faculties during the 1790s, while still in Vienna. Over the next two decades, he would keep modifying his tentative lists, the names he gave to his fundamental faculties, and the parts of the brain he believed housed the organs critical for each function.

Often overlooked, Gall’s earliest known lists included four brainstem faculties (Finger and Eling, 2019). Only later did he take his medullary and midbrain faculties off them, limiting his system to the brain’s outer rind, its cortex. What he never did do, however, was to restrict his functions of mind to the cerebral cortex. Rather, he opted to retain one faculty in the *cerebellar* cortex. This was the faculty he associated with a higher-order (e.g., conscious, not entirely reflexive) sort of reproductive drive or instinct in humans and higher animals, and it became the first and most primitive faculty on his final list.

In his two sets of volumes, his *Anatomie et Physiologie du Système Nerveux*… from 1810 to 1819 and his less expensive *Sur les Fonctions du Cerveau…* of 1825, in which he dispensed of his detailed neuroanatomy while retaining what he sometimes called his *organologie*, Gall described his chosen 27 faculties in great detail. His list starts with his most primitive faculty of mind and works its way up to what he considered eight distinctly human faculties. Importantly, he placed the organs for our highest faculties, such as those for wit and religion, in the top of the front of the brain, while assigning successively more primitive functions to brain organs located more posteriorly. Hence, reproductive instinct, the first and most primitive item on his ascending list, was the function he assigned to the cerebellum.

Gall could be very dogmatic, but he acknowledged when coming forth with what would become his final list that there could well be overlooked faculties with territories not yet discovered, especially since the skull does not faithfully reflect the morphology of the lower side or underside of the brain. In effect, he told his readers that his system should be regarded as a work in progress: an ongoing project open to new evidence that might require modifying the list. Gall was far more certain about the basic tenets of his doctrine than his chosen faculties and the sites of their organs. His concepts, such as cortical localization and innate, independent functions, he believed, would prove far more enduring than the 27 faculties he tentatively settled on, each dependent on localized pieces of tissue on the two sides of the brain.

## Reports from Vienna on Gall’s Faculties and the Role of the Cerebellum

Ludwig Heinrich Bojanus (1776–1827) was born in Bousville (Buchsweiler), Alsace, but after the French Revolution of 1789, when the French Army reconquered Alsace, the Bojanus family left and settled in Darmstadt, where Ludwig completed his secondary education. He then went to the University of Jena to pursue his medical career, graduating in 1797. Bojanus next traveled to Vienna, where he practiced in the General Hospital during 1797–1798, attended some of Gall’s lectures, and probably also discussed some of Gall’s ideas directly with him before leaving the city with extensive notes about the German physician and his novel doctrine.

Bojanus provided two early accounts of what Gall was covering in his Vienna lectures, one in French in 1801 and the other in English a year later (Bojanus, 1801, 1802; Sakalauskaitė-Juodeikiene et al., 2017, in press). These articles were titled “Encephalo-Cranioscopie. Aperçu du Système Craniognomíque de Gall” (“Encephalo-Cranioscopie. Overview of the Craniognomic System of Gall of Vienna”) and “A Short View of the Craniognomic System of Dr. Gall, of Vienna.”

He began his list of Gall’s faculties with the most primitive, which was how Gall presented them in his lectures, and they included some that did not involve the cerebral cortex. He explained that Gall contended that the medulla oblongata is the seat of the organ responsible for tenacity of life, since “there are no speedier means of killing an animal than to cut the medulla oblongata” (Bojanus, 1802, p. 81). Further, he tentatively placed self-preservation “a little further forward in the medulla oblongata,” although he felt somewhat unsure of the locus and was evaluating more evidence for it (p. 81). Additionally, he localized the organ for choice of nourishment in the quadrigemini tubercles (colliculi), since the anterior tubercles seemed larger in carnivores, the posterior tubercles seemed bigger in graminivores (literally “grass-eating” or herbivorous animals), and these structures were of equal size in omnivores.

After localizing the cerebral organs of the external senses (number 4 on his list) in the middle region of the base of the brain, Bojanus related that he then turned to the organ of instinct and copulation, which he opined is dependent on the integrity of the cerebellum under the base of the occipital bone. He explained that “this organ never expands but at the age of puberty, and that its increase has a great influence on the form of the nape of the neck, because to this part of the cranium its muscles are affixed” (p. 82). Further:

In animals, castrated before the age of puberty, the expansion of this organ does not take place.

In the ape, the hare, and the cock, this organ is very apparent; and in pigeons and sparrows the occipital forms a particular bag which seems to be an appendage of the head; and it is well known that these animals are exceedingly ardent in copulation. The same disposition is sometimes found in the cranium of man; and Dr. Gall has in his collection the skulls of several fools, who were distinguished by their lasciviousness, and whose occipital bone forms an enormous projection (p. 82).

Bojanus was not the only man of medicine to report on Gall’s system as it was taking shape in Vienna. Ludwig Friedrich Froriep (1779–1847) also met Gall while in Vienna. Having finished his basic medical education in Jena, he was on a study trip in 1799 when their paths first crossed. He would subsequently accompany Gall when he visited the prison in Torgau to gather more cases for his emerging doctrine. But that would be 6 years later, in 1805, when Gall had started on his lecture tour through the German states, beginning in Berlin.

Froriep had already published an article on Gall before Bojanus’ first article appeared, but because he attended Gall’s lectures about a year after Bojanus did, what he wrote about Gall’s developing doctrine is presented second here. In fact, Froriep had three publications, one in 1800 and two in 1802 (Froriep, 1800, 1802a,b). The most important was his 1802 book titled *Darstellung der ganzen, auf Untersuchungen der Verrichtungen des Gehirns gegründeten, Theorie der Physiognomik des Dr. Gall in Wien* (*Account of the Entire Theory of Physiognomy of Dr. Gall in Vienna Based on Investigations of the Brain*; Froriep, 1802b). This work circulated widely.

Froriep opened his account with Gall’s guiding principles. He then described his faculties under the heading *Stufenleiter der Veredlung der Thiere* (*Ladder of Ennoblement of Animals*). This was Gall’s version of the Great Chain of Being, sometimes represented as steps or with a ladder. On the lowest step involving animals, he included those representing the transition from plants to animals, creatures like polyps, which lack a real nervous system and remain stationary. On the second step, he placed animals with a spine and primitive brain that are capable of moving and sensing. Here he mentioned worms, which will die if the spine is cut off from the brain. Froriep explained that these kinds of observations led Gall to conclude that the *Organ des Lebenskraft* (organ of vital force) must be located in the medulla oblongata. At a still higher level, Gall included animals that reproduce through copulation. These animals exhibit two clear knots that form the *Organ des Begattungstriebes*, meaning the organ of instinct of copulation, just above the spinal cord. These knots would become the cerebellum in more advanced animals, including humans, and the structure critical for a conscious and controllable sort of reproductive instinct. Gall then directed his attention to successively higher functions and their associated organs, all of which he associated with the cerebral cortex.

Philipp Franz Walther (1782–1849), born in the small German village of Burrweiler, some 30 miles (50 km) northwest of Karlsruhe, was a third man of medicine to publish what Gall was relating in his Vienna lectures during the opening years of the 1800s. Walther studied medicine in Vienna but obtained his medical doctorate from the University of Landshut in 1803. A year before receiving his degree, he published his *Critische Darstellung der Gall‘schen anatomisch-physiologischen Untersuchungen des Gehirn- und Schädel-baues* (*Critical Account of Gall’s Anatomical–Physiological Investigations of the Brain and the Form of the Skull*; Walther, 1802).

Walther criticized Froriep’s renditions, considering them no more than loose notes that failed to meet his own high standards. Not surprisingly, given how these authors drew from the same sources, the ground plan of Walther’s book is similar to that of Froriep and Bojanus. Following Gall’s coverage from one lecture to the next, he too began with a discussion of basic principles and then worked into descriptions of the individual faculties of mind, starting with the most primitive and later discussing those that only humans display, along with the evidence Gall gave for each and its organ.

Thus, Walther (1802) began his tour of Gall’s faculties with the *Organ der Lebenskraft* (organ for the vital force), which he localized in the oblong medulla. Nonetheless, Walther did not think this was the best name for this organ and its presumed function, although he could not come forth with a better name. From this part of the lower brainstem, he moved on to the minor or small brain (*Halbkugeln des kleinen Gehirnes*). One can already detect this structure in the insects, Gall related, but not in their larvae: it only develops with the genital organs during the “flower season,” a reference to the warmer months of spring and summer. Quoting Walther, who was quoting or paraphrasing Gall: “The size of the cerebellar hemispheres is certainly in proportion to the level of activity of the sexual drive; this can be shown throughout the animal kingdom” (Walther, 1802, p. 79–80).

Dutch anatomist Gerardus Vrolik (1775–1859), who taught at Amsterdam’s Athenaeum Illustre, followed 2 years later, publishing his *Het Leerstelsel van Joseph Gall* (*The Doctrine of Joseph Gall*) in 1804, a year before Gall left Austria on his European lecture tour (Vrolik, 1804). Vrolik differed from some of the others publishing early accounts of Gall’s theory, in that he was already an acknowledged expert on brain anatomy and comparative anatomy. He also possessed a collection of about 5,000 objects that included bones, newborns with abnormalities, and skulls from people who lived all over the world.

Vrolik’s book was based on two lectures he gave at the Felix Meritis [Happiness via Merit] Society in Amsterdam at the beginning of 1804. He agreed with Gall that brain organization can be related to mental capacity, and he praised him for recognizing that each faculty must have its own organ—an organ that develops on its own and is functionally independently of other organs. He then discussed Gall’s faculties of mind, which he stated now numbered 30.

Gall’s first faculty again dealt with the vital force, which he was still associating with the oblong medulla. His next one was self-preservation. Vrolik stated that Gall still seemed uncertain about its exact locus in the brain, but thought it must be located at a higher level than the medulla immediately above the spinal cord. Gall’s third organ, he continued, determines the choice of food. It can account for distinctions between herbivorous, carnivorous, and omnivorous animals, and it is located in the anterior and posterior tubercles. He called the next organ on Gall’s list *teeltdrift*, which loosely translates as the “drive” for propagation, and related that it is dependent on the cerebellum. Thus, Vrolik’s description is comparable to that of Bojanus and some of Gall’s other listeners, although he used old-fashioned Dutch terminology and wrote less clearly.

Dutch physician Jacob Elisa Doornik (1777–1837), Vrolik’s colleague at the Athenaeum Illustre, published his own book on Gall and his doctrine in the same year as Vrolik (Doornik, 1804). It bore the title *De Herssen Schedelleer van Frans Joseph Gall getoetst aan de Natuurkunde en wijsbegeerte* (*Franz Joseph Gall’s Theory on the Brain and the Cranium, Tested by Physics and Philosophy*). He was less than kind to Gall and criticized him for not providing ample evidence for his bold pronouncements, for being materialistic, and for drawing tenuous conclusions.

In the third section of his book, which covered the brain as an “aggregation” of organs for different mental capacities, propensities, and impressions that can be discerned from the skull, Doornik discussed each of Gall’s organs, again adding critical remarks. He too began with the organ of vital force, situated in the medulla oblongata, but wrote that Gall localized the organ of lust or sex (in German, *Geslechtstrieb*) not far away from it, in the cerebellum. Interestingly, Doornik, like Vrolik, chose words suggesting that Gall viewed the reproductive faculty more like a drive than an instinct.

Next to the uppermost part of the oblong medulla and the egg-shaped hole, two extensions of the lower part of the cerebellum present themselves, these form the organ of sexual activity. This organ is, like others, double. When it is developed to a remarkable degree one can find larger bulges at the lowest part of a skull, and smaller bulges if it is developed less. During infancy, this development is zero (“= 0”), and it expresses itself only at a marriageable time. This organ, if developed excessively, causes horny lust (Doornik, 1804, p. 164).

Andrew (or Jędrzej) Sniadecki (1768–1838) provided our last example of where Gall stood on the cerebellum prior to leaving Vienna. Sniadecki was born in Żnin, in the Polish–Lithuanian Commonwealth (Sakalauskaitė-Juodeikiene et al., 2017). After completing his medical studies in Vilnius, he worked in the Vienna hospitals and became acquainted with Gall and his ideas. He then returned to his homeland to become professor of natural sciences at Vilnius University.

While in Vilnius, Sniadecki informed students, physicians, and others about Gall’s new doctrine, and wrote an article about it that was published in 1805. He chose “Krótki Wykład Systematu Galla z przyłączniem niektórych uwag nad iego Nauką” (“Short Lecture on the System of Gall With Some Comments About his Science”) as his title, and it appeared in the journal *Dziennik Wilenski* (see Sakalauskaitė-Juodeikiene et al., 2017). In it, he first mentioned two organs related to life itself: an organ of vital force and an organ of binding (tenacity) to life. He stated that Gall localized the former in the posterior medulla oblongata, near the end of the spinal cord, and the latter near the foramen magnum. Sniadecki then went on to the organ of lustfulness (also referred to as amativeness in other accounts) and how Gall localized it in the cerebellum.

## The Cerebellum During Gall’s 1805–1807 Lecture Tour

Thus, Gall associated the reproductive instinct or drive with the cerebellum before he left Vienna in 1805. We shall now see that he remained firm on this association while he lectured in various European cities and after he settled in France in 1807. We begin with an 1805 publication by Christian Heinrich Ernst Bischoff (1781–1861) to make this point.

Bischoff was born in Hanover, and he obtained his medical degree in Jena, in 1801. In 1804, he was appointed extraordinary professor of physiology in Berlin, where he had assisted his mentor and now friend, Christoph Wilhelm Hufeland (1762–1836). Bischoff attended Gall’s first series of lectures on his scientific tour, those given in Berlin, where to his delight, he was warmly received.

Since Gall had not published anything about his organology (Gall never used the term *phrenology*, which was only introduced in 1815 in England) since his 1798 letter to Retzer (Gall, 1798), and given the dates and quality of some of the publications by others, Bischoff recognized the need for an informative, accurate, and more up-to-date publication. He titled his book *Darstellung der Gall’schen Gehirn- und Schädel-Lehre* (*Account of Gall’s Brain and Skull Theory*; Bischoff, 1805). Two years later, this work was edited, expanded, and translated into English (Bischoff, 1807).

Gall had removed his earlier brainstem organs for a vital life force, binding to life, and nourishment from his list when Bischoff heard him lecture in 1805. He might now have viewed the life force as too metaphysical and also reasoned that these three faculties could not really be classified as functions of the mind. His revised list now started with the *Organ der Geschlechtsliebe* in the cerebellum, which was called “the organ of sexual love” in the 1807 English translation of his book.

In contrast to earlier writers, Bischoff provided more of Gall’s evidence for this structure–function association, writing:

It has already been observed that as sexual passion arises, this part of the brain (the cerebellum) grows in disproportion to the other parts (the cerebrum); and when, by castration, the purposes of nature in the formation of this organ are defeated, we find that this organ ceases to develope [sic] and perfect itself. It is observable in all who have suffered this operation when young, that the back of the skull, as it were, ceases to grow; the neck is narrow, and the voice, whose seat is in the throat, loses its manly vigor (Bischoff/Hufeland/Robinson, 1807, p. 81).

Moreover, and with regard to animals:

This remark is equally made in many species of animals…. The stallion and bull have a more perfectly developed cerebellum, and consequently have a thicker neck and broader head behind, than the gelding and ox. This is known to the common people who are concerned in the breed of horses, who give preference to those stallions whose ears stand the widest apart. The male mule, which has no power of procreation, generally speaking, has a very narrow neck, and the ears stand close together…. Throughout the whole class of quadrupeds, the neck of the male is thicker than that of the female. Gall attributes this to the longer duration of the sexual appetite in the male (pp. 81–83).

And for additional evidence, there were notable pathological conditions:

In nymphomania, Gall has found the neck very hot, swollen, and painfully inflamed.

Wounds in the neck and back of the head will produce inflammation of the parts of generation and even impotence.

*Cretins* are notorious for their lasciviousness, while they are without the common intellectual powers, and their cerebellum is unusually large. The known effects of sleeping on the back, Gall also attributes to the pressure and warming of the cerebellum.

Among other cases of insanity, G. related one of a man, from whom the fixed idea could not be removed that he had six wives, &c. The cerebellum was found monstrously large after his death. Once, on entering an hospital, in which he never was before, he heard a mad woman uttering the grossest obscenities, he desired the attendants to go and examine her head, declaring that if they did not find the skull remarkably large behind, he would renounce all his opinions. He was not deceived (pp. 83–84).

Gall, he related, also cited artistic renderings to show how firmly the reproductive drive could be connected to the cerebellum.

The bust of Raphael which was made from an impression taken in Gypsum, exhibits a sort of bad behind, announcing that tendency of his constitution to which he unhappily fell and early victim (p. 84).

Although this comment about Renaissance painter Raphael Sanzio da Urbino (1483–1520) closed Bischoff’s section on the reproductive faculty and organ, he pointed out that Gall’s second faculty and cortical organ were closely related to his first organ. Gall was calling his second organ the *Organ der Kinder- und Jungenliebe*, and it is presented as “the organ of parental and filial love” in the English translation. It is the closest organ physically to the organ of sexual love, having a posterior occipital cortex site. Gall would emphasize that unlike his first organ, which is usually dominant in males, love of offspring is typically stronger in females.

Martinus Stuart (1765–1826) provided a second account worthy of note, and it appeared a year after Bischoff’s book. Stuart (1806) titled his contribution from the Netherlands *Herinneringen uit de lessen van Frans Joseph Gall* (*Memories from Franz Joseph Gall’s Lessons*). Unlike the authors previously mentioned, he had studied theology, served as a minister, and was regarded as a very good historian when he set forth to describe each of the 10 lectures Gall had given in Amsterdam.

Gall presented his “preliminaries” in his first five lectures, holding back until the sixth to start working through his list of faculties. His first one is labeled *Geslachtsdrift* in Dutch, which would signify sex *drive* in English, much like Vrolik’s term *teeltdrift*. As before, the organ for this faculty was localized in the cerebellum.

What is also memorable in Stuart’s rendition is how he stated that, just before turning to the cerebellar organ, Gall had related that large lesions of the oblong medulla lead to immediate death. This was something noted in other earlier accounts. But, Stuart continued, Gall was now contending that medullar lesions should not be considered as proof of a special organ for *Levenskracht*, the vital force. He stated Gall was, in fact, maintaining that he had never accepted such a force, although others had written that he had previously made this assertion. He continued by clarifying Gall’s position, which was devoid of a metaphysical force and based on the contention that the oblong medulla is merely the connection between organic (*werktuiglijk*) life and animal life. Removing this connection ends “life-consciousness,” including voluntary control over body parts, while the organs still remain intact.

Thus, Gall was listing the instinct (or perhaps better, drive) for propagation first among his faculties of mind as he made his way into the Netherlands in 1806. The fact that he was now clearly dismissing the vital force (*Lebenskraft)* as a faculty of mind is consistent with how he had been rejecting metaphysical forces and abstract capacities or faculties, two features that had dominated the literature on the mind, soul, and brain since classical times. The traditional view had been that life involves a spiritual force or power that enables movement, and prior to the 1800s, “vitalism” had permeated virtually every aspect of natural history as well as broader culture. Physician Georg Ernst Stahl (1660–1734), for one, had played a prominent role in the promotion of vitalism in physiology during the 18th century. In contrast, Gall had begun to make the case for dismissing every trace of metaphysics from medicine, including Stahl’s still-highly-influential views, in his first book, published in 1791. In hindsight, what is somewhat surprising is that (at least according to his listeners) he even bothered to mention *Lebenskraft* and relate it to the medulla in his earlier lectures.

## Gall’s Own Detailed Descriptions

This brings us to how Gall described the function or functions of the cerebellum in his own writings on organology, that is, the books that began to come off the press in 1810. With regard to what follows, it is important to remember that he transferred what he had written about the faculties in his *Anatomie et Physiologie du Système Nerveux…* to a less expensive set of books that he published in 1825, his *Sur les Fonctions du Cerveau…*, merely adding several retorts to his critics and bits of additional supportive evidence, while leaving his basic ideas and associations intact. The 1835 English translation of the latter volumes, his *On the Functions of the Brain and Each of its Parts…*, provides a faithful translation of the latter volumes (see Finger and Eling, 2019). Additionally, George Combe’s (1788–1858) and his brother Andrew Combe’s (1797–1847) *On the Functions of the Cerebellum…* from 1838 contains an English translation of Gall’s French text on the instinct of propagation, along with a few paragraphs showing how he responded to his critics at a later time, and we will quote from it.

In his major publications, Gall’s first two faculties are presented as: (1) instinct of generation, of reproduction, instinct of propagation and; (2) love of offspring. Without question, Gall considered the continuation of the species to be primary on his list of faculties, and to him, reproduction and caring for offspring were two closely related, basic functions. Reflecting this, he provided more information about them and their associated cortical organs than he did for any of his other faculties.

Gall’s organ for propagation, however, remained unique. Unlike his other 26 organs, it never had a cerebral cortical location. Rather, he continued to retain it in the cerebellum, where it remained separated from his organ for love of offspring, which, as befitting organs having much in common, was localized nearby—in this case, in the posterior occipital cortex.

Gall began his section on the reproductive faculty by claiming that the instinct of reproduction is a faculty of the brain and not of the sexual organs. He would make similar statements with respect to some of his other faculties, such as his faculty for tune or music, which he argued is not a faculty for simply hearing sounds or one based in the ears. He repeated the same thought when discussing his faculty for color relationships, which he contended is involved with perceiving color harmonies or relationships and is by no means housed in the eye. What Gall wrote about his chosen faculties being higher-brain and not peripheral functions might seem obvious to us today, but this was not always the case in the past. In fact, we still use expressions like “he has a good ear” or “she has a good eye” when describing someone able to recognize the intricacies of a concerto or an oil painting, reflecting what many people living in earlier times believed.

**Figure 2:**
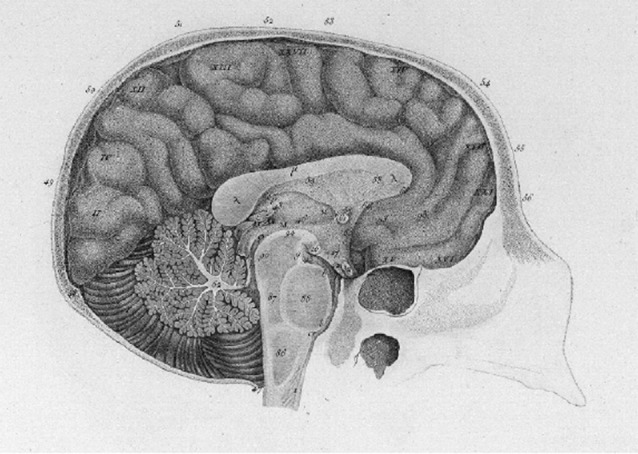
Figure from Gall’s 1810 atlas (p. 10) depicting the instinct of generation, of reproduction, instinct of propagation (I) in the cerebellum.

Gall was interested in describing the interactions that could occur between the brain and the sexual organs, and in his “great work” and its less expensive 1825 edition, he discussed the influence of the brain on the reproductive organs and *vice versa*. Deviating from the descriptions of most of his other faculties, where he introduced the part of the brain or organ associated with a given faculty at the end of his description of the faculty, its history, and the evidence for it being independent, he opted to point to the cerebellum as the material condition or seat for the instinct of reproduction early on in its chapter (see Figure 2). As far as he was concerned, this was a hard fact.

When presenting human and animal evidence to support his conclusions, Gall cited a woman he had personally seen, providing these details:

A young widow, a short time after the death of her husband, was attacked by melancholy and violent convulsions. These affections were preceded by a very disagreeable tension and feeling of heat in the nape of her neck. A few moments afterwards, she fell to the ground in a state of rigidity, to such an extent that at last the nape of her neck and vertebral column were strongly drawn backwards. The crisis never failed to terminate by an evacuation, which was accompanied by a convulsive voluptuousness, and a real ecstasy; after which she continued free from attacks for some time.

I frequently supported her during the crisis with the palm of my hand on the nape of her neck, and felt at that spot a great heat. I remarked also an arched protuberance of considerable size in the same place. Afterwards this lady acknowledged to me, that from the close of childhood, she had felt it impossible to resist the impulses of an imperious necessity; and that in these moments, when her feelings were the most urgent, the tension and sensation of heat in the nape of the neck were the most disagreeable (Gall, 1838a, pp. 12–13).

He also brought up cases mentioned by others. For example, he alluded to observations by Apollonius of Rhodos (c. 295 BC–c. 215 BC), Jacob van der Haar (1717–1799), and Samuel Tissot (1728–1797), further suggesting a relationship between convulsions, emissions, and pain in the neck. And when pointing to the cerebellum as the site of the organ critical for the reproductive instinct, he noted how the size of the cerebellum differs significantly across individuals, as does the reproductive drive.

Gall knew there were anatomists who were questioning and even denying the possibility of determining differences in the size of the human cerebellum by examining skulls or other overt features. He argued, however, that he could establish the size of the cerebellum by measuring the distance between the mastoid processes. If the cerebellum is large, he opined, “the nape of the neck is large and thick, the neck rounded, and large and thick behind the ears” (p. 15). In other words, although dissections would be ideal, they were not necessary, at least when it came to estimating the relative sizes of cerebellums.

As revealed in Bischoff’s report, Gall liked to present many different kinds of evidence to bolster his impressions and conclusions. With regard to the reproductive instinct, some of his evidence was positive and some negative; some from animals and some humans; some drawn from studying healthy brains and some from sick or damaged brains; some from comparing males to females; and more. When possible, he would also compare people of different ages, Europeans to people from far-off places, and individuals of the same nationality but from different climates. To his credit, Gall fully appreciated what scientists today call “the power of converging operations,” and he applied this strategy of bringing together many different kinds of “evidence,” some better than others, especially when discussing the faculty and organ for the reproductive drive or instinct. Nonetheless, he showed more faith in cranioscopy than any of his other methods, and without question, he was led astray by this decision and by how readily he dismissed things that did not support the biases stemming from his cranioscopy.

In his books, Gall began his support for the cerebellum being critical for the reproductive faculty with “proofs drawn from the state of health” (p. 16). First, he wrote, nothing resembling a cerebellum can be distinguished in animals that propagate without conjunction of the sexes. In contrast, animals that copulate have cerebellums. Here Gall made the very important distinction between fecundation and the inclination to sexual intercourse. He maintained that the existence of fecundation without the intervention of the brain cannot be interpreted as proof that conscious reproduction can be accomplished without a brain. When he listed the reproductive instinct or drive as a faculty of mind, and particularly when discussing humans, he had consciousness, choice, and other features of a higher “mental” function very much on his mind.

For another sort of evidence, he compared “mammiferous” and oviparous animals with respect to both how they propagate and the structure of the cerebellum: “In oviparous animals and insects, fishes, and amphibious creatures,” he explained, “the fundamental portion constitutes the whole of the cerebellum,” whereas “[i]n all the mammiferous animals, on the contrary, the two lateral portions exist” (p. 18).

Third, he pointed out that “the successive stages of increase and decrease in the manifestation of the sexual instinct, bear a direct relation to the increase and decrease of the cerebellum” (p. 18). Gall mentioned Samuel Thomas Soemmerring (1755–1830), this great anatomist’s student, Jacob Fidelis Ackermann (1765–1815), and Joseph Wetzel (1768–1810) and his brother Karl (1769–1818) in this context, but all negatively. They maintained that, by the age of 2 years, the cerebellum is fully developed. Gall disagreed, contending that this was not in accord with his own observations. He pointed out that humans can display rather astonishing individual differences in this faculty and its organ. He had personally observed a 5-year-old boy, who behaved more like a 16-year-old man, and he also mentioned a girl of 9 years, who acted as if she were a mature woman. These individuals were outliers to him, since the reproductive drive in humans, which is dependent on cerebellar development, typically comes of age during the teenage years.

His fifth argument involved the relationship between the “energy” of the reproductive instant and the development of the cerebellum. “There are men and women who perform the act of cohabitation only as an act of duty,” he noted, and “they uniformly exhibit a feeble development of the cerebellum” (p. 23). Gall provided several “case histories” to support this claim, adding that a similar pattern can be found in animals, providing concrete examples.

His next line of evidence involved gender differences. “The difference observable between the two sexes in regard to the degree in which they manifest this instinct,” he related, “depends also on a difference in the degree of development of the cerebellum” (p. 28).

And his seventh argument was further revealing of behavioral differences that could be seen by studying animals. In his words

The kind of caresses which certain animals practice, should have long ago arrested the attention of naturalists. Sometimes the male, sometimes the female, is in the custom of exciting the nape of the neck of the objects of their desires. Long before the act itself, the male cat bites, amorously, the nape of the neck of the female (p. 32).

With this statement, he concluded his proofs pertaining to healthy people and animals. But this was only one line of evidence. He now turned to what he had learned by examining people and animals with naturally occurring disorders, acute injuries, and surgical procedures, starting with what could be observed after castration.

Castration, he knew, could be performed at an early age or later in life, both in humans and animals. Nonetheless, its effects are more dramatic in the first case, including those affecting the cerebellum. “It is from this defect in the development of the cerebellum, and not at all from the inferior prominence of the muscles,” he explained, “that all castrated animals have the nape of the neck thinner and narrower than entire animals, such as the ram, the bull, &c” (Gall, 1838a, p. 37). He continued: “This imperfect development of the cerebellum is also the sole cause why, in some cases, the instinct of propagation does not manifest itself, or manifests itself only in a very imperfect manner” (Gall, 1838a, p. 37).

The crucial role of the cerebellum in the development of a normal functioning organ for propagation is further supported by the fact that castration after development has taken place does not result in such marked effects. “When castration takes place after the completion of growth, or at least at a period when the cerebellum is already to a great extent developed,” he wrote, “it does not prevent the manifestation of the instinct of propagation, nor destroy the power of exercising copulation” (pp. 37–38).

Gall now argued,

[T]he effect produced by the removal of a single testicle is undoubtedly the most decisive. On every occasion when one testicle only has been removed from any animal, of whatever species, the lobe of the cerebellum, on the opposite side, visibly decays, or is altered in some way in its substance (p. 40).

Not only castration but also other conditions affecting the sexual organs, can have notable effects on the cerebellum. He cited hunters, who from earlier times were recognizing that injuries to a roebuck’s testicles could result in deformations of its horns. He also described how humans with testicular injuries have diminished cerebellums.

He then turned things around and explained how damage to the cerebellum could affect the genital organs. He began with some ancient history, stating that Hippocrates (c. 460–c. 370 BC) taught that “behind the ears, there are veins, the section of which produces impotency; and it is precisely in those, I believe, that they bleed themselves, for, when they approached their wives, they found themselves incapable of executing the act of copulation” (Gall, 1838a, p. 45). He then mentioned cases he had personally observed or that had been observed by others, in which brain injuries caused impotency, some with postmortem observations revealing cerebellar damage.

Gall treated diseases of the cerebellum and their effects on the genital organs separately from acute injuries. He knew that some diseases could irritate genital organs yet have no effect on the instinct of propagation. He mentioned a man stung by an insect, who had violent erections without any voluptuous sensations (Gall, 1838a, p. 56). In contrast, there was another young man living in Vienna, who suddenly fell into a state of erotic mania with erections and pain in his testicles. Gall related how physicians looked for local inflammations in this young man’s genital organs but failed to find the cause of his erotic mania situated there. He was then called in to examine this patient, and he suggested that his condition might be the result of a cerebellar problem. He designed a treatment based on this assumption, and in a few days, the man’s mania diminished. He also discussed a case seen by Philippe Pinel (1745–1826) and another conveyed by Jean-Étienne Dominique Esquirol (1772–1840). Both cases had erotic manias, and both exhibited physical features he associated with abnormally large cerebellums.

Still another category of his pathological proofs consists of “observations of the activity or inactivity in Idiocy.” Here he wrote:

I have examined a great number of these individuals, and the following is the result of my researches. Whether the genital organs be large or small, they never exercise a determinate influence on this instinct. The propensity remains inactive in all cases in which the cerebellum has attained only to a small degree of development (p. 66).

Gall brought up the wild boy or savage of Aveyron in this context. As a feral child discovered in 1800, when he was already 12 years old, Victor (1788–1828) had shown minimal interest in women. Based on skull features, Gall contended, his cerebellum appeared to be only weakly developed.

He now brought excessive mastication into the picture. “In several hospitals for the insane, and in some houses of correction, we have met with subjects who were said to have become insane in consequence of excessively frequent emissions of the seminal fluid, or who were devoted to punishment for having given themselves up to onanism” (Gall, 1838a, p. 68). Gall did not want to suggest that masturbation would invariably lead to a defect in intelligence. Nevertheless, he did want to direct attention to the “fact” that the faculties located in the anterior part of the brain tend to be poorly developed, while those located in the posterior part are typically well developed in these cases. Consequently, control of the lower-order faculties will be limited in these people, making them comparable to an “ape in heat” (p. 68).

In his next paragraph, Gall argued, “The Instinct of Propagation survives the destruction of the Genital Organs, and exists in the absence of these parts” (p. 69). Women with their genital organs impaired because of tumors or other disorders might still desire to make love. Similarly, he mentioned how he had already spoken of the instinct of propagation remaining after the testicles have been removed, as well as when, as he put it, “the functions of the womb had completely ceased” (p. 71). His point bearing repetition is that he is describing a brain function and a higher, cortical one at that.

Gall now turned to “particular diseases of the cerebellum.” In his books in French, he mentioned only one patient, an individual he had personally observed in Vienna. But in the Combe and Combe (1838) English translation, he also brought up cases from several esteemed French physicians, including Étienne Serres (1786–1868), Frédéric Dubois (1797–1873), and Jean-Pierre Falret (1794–1870). Each of their cases, he claimed, confirmed the points he was making.

Gall’s last group of disorders in this translation involved different forms of apoplexy that he believed affected the cerebellum. This category also cannot be found in his books. Yet it seemed to have been written by Gall, not differing stylistically from what he wrote in his French descriptions of the cerebellar organ and its special function, and supporting his earlier claims.

After discussing diseased states of the cerebellum, Gall formulated a series of conclusions and implications. Because of their importance, we have chosen to quote him rather than to paraphrase or summarize what he wrote.

These numerous physiological and pathological facts observed in man and the lower animals, not only prove that the cerebellum is the organ of the instinct of reproduction, but they serve also to explain the following phenomena: —

1. How irritants applied to the nape of the neck, such as blisters, setons, frictions with volatile and spirituous substances, often produce a violent irritation in the genital organs, excite the menstrual discharge when it has been suppressed, remove complaints caused by its suppression, and cure impotency arising from debilitating causes, much better than all the means which are usually made to act on the sexual organs.
2. Why, on the contrary, cupping-glasses, leeches, cold lotions, and embrocations, applied to the nape, frequently cure erotic mania, especially when it has appeared suddenly, and constitute excellent remedies against priapism, satyriasis, nymphomania, and nocturnal pollutions, always assuming, however, that these last are not a consequence of exhaustion.
3. Why hanged men have violent erections and abundant emissions of seminal fluid. If it be true that the same symptoms manifest themselves in furious madness, frequent bleedings, &c., in the nape, might perhaps produce beneficial effects in this disease also.
4. Why, in some injuries of the brain, the wounded direct their hands first to the organs of sex, and then to the head.
5. Why, in the case of inflammation of the genital organs, there is always a great danger when delirium and inflammation of the parotid glands is combined with it, or when, in delirium, the patients often direct their hands to the sexual organs.
6. Why the disease terminates almost always in death, when in these cases there is delirium, disordered and convulsive movements, and prostration of strength; symptoms which are usually explained by a typhus fever, while they proceed from an inflammation of the brain.
7. Why, in men who have died of apoplexy produced by the efforts of a voluptuous coition, we almost always find blood effused in the cerebellum. Very lately I have had a fresh opportunity of confirming this observation.
8. Why excessively ardent amorous desires are frequently the precursors of apoplexy.
9. Why a very ardent copula, too frequently repeated, is capable of producing mental alienation. Forestus, lib. x., observ. 25, reports an example of this occurrence.
10. Why, in the Turkish and Persian soldiers, who have made an excessive use of opium, erections continue a long time even after death (pp. 92–94).

## Flourens Vs. Gall on The Cerebellum

**Figure 3:**
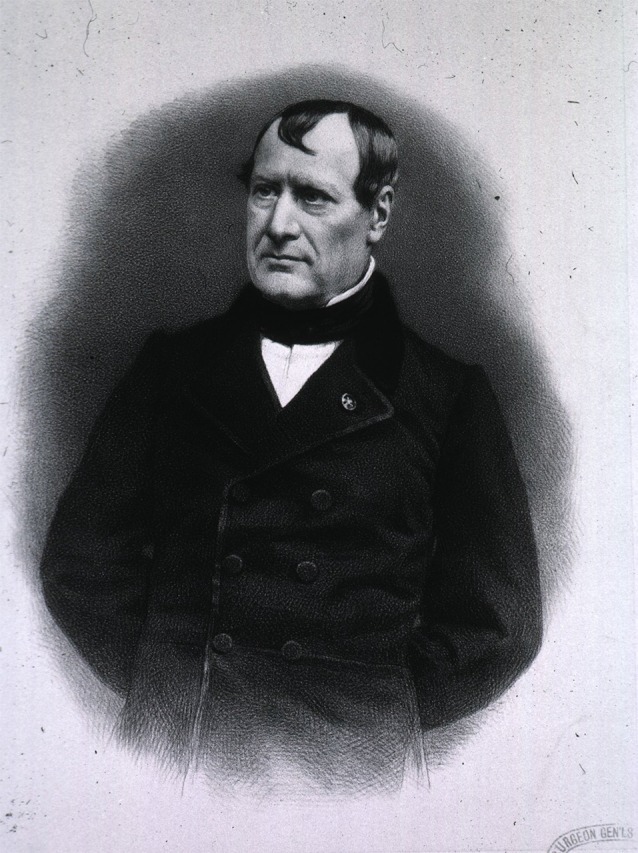
Jean Pierre Marie Flourens (1794–1867; picture from the public domain).

Shortly after Gall had finished the final volume of his *Anatomie et Physiologie…*, a young French physiologist started a series of experiments on the irritability of several nervous structures. This young man was Jean Pierre Marie Flourens (1794–1867; Figure 3). He was born in the village of Maureilhan (near Béziers) and had been sent to Paris, where he studied and worked under physician Étienne Geoffroy Saint-Hilaire’s (1772–1844) supervision. With his guidance, Flourens examined Albrecht von Haller’s (1708–1777) views on the irritability of the nervous system by conducting lesion experiments on various animals. He did not set forth to test Gall’s concept of localization of function, as so often has been asserted, and even seemed to admire Gall’s anatomy and his way of thinking when he began this research (for more, see Finger and Eling, 2019).

**Figure 4:**
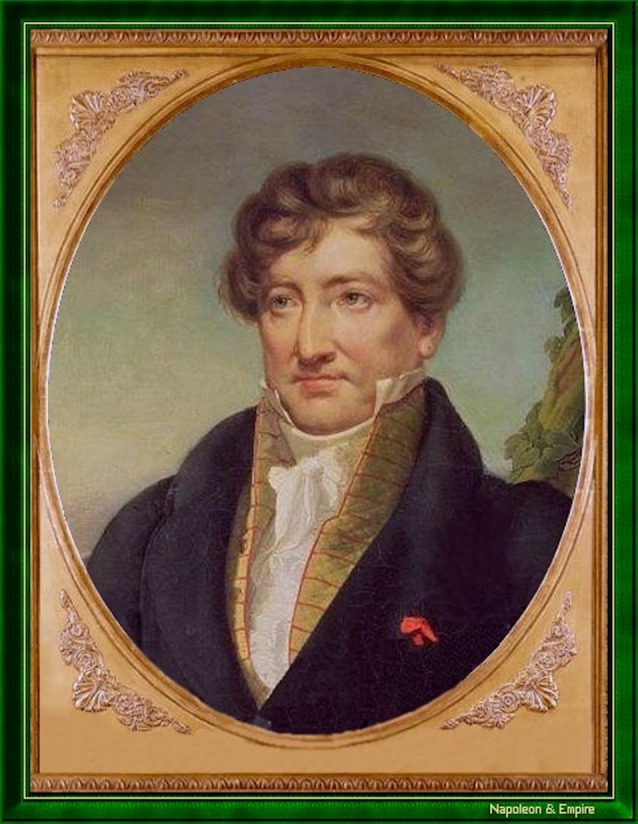
George Cuvier (1769–1832; picture from the public domain).

The findings stemming from Flourens’s experiments were first reported by George Cuvier (Figure 4), who had rejected Gall and Spurzheim’s submission to the *Académie des Sciences* in 1808 and then continued to be put off by how Gall fought back in his letters and in public. Flourens had posed three questions, which the senior scientist, Cuvier, presented in 1822 in a *Rapport to the Acad*é*mie* that was published 2 years later (Flourens, 1824, p. 68).

1. From what points of the nervous system might artificial irritation depart, so as to reach the muscles?
2. To what points of this system must the impression be propagated to produce sensation?
3. From what points does voluntary irritation descend, and what parts of the system must remain entire to produce it regularly?

Flourens had “defined” the nervous system as the system involved in receiving sensations and producing movements. He was especially interested in movements and distinguished between involuntary and voluntary ones while recognizing the will as a mental faculty. It was with this mindset that he had examined the effects of damaging different parts of the nervous system in animals. These parts included the cerebellum, corpora quadrigemina, medulla oblongata, and spinal cord.

Flourens (1824) claimed that earlier investigators had failed to demonstrate the specific functions of the parts he was studying. The biggest problem stemmed from the methods previously used. Applying irritating chemical substances, electricity, or pressure to the brain made it impossible for them to distinguish between the local and distal effects of what they were doing. He specifically singled out Haller and his student Johann Gottfried Zinn’s (1729–1757) many experiments, along with those of fellow Frenchmen Anne-Charles Lorry (1726–1783) and Nicolas Saucerotte (1741–1814). Hoping to come forth with more easily understood findings, Flourens turned to lesion experiments on birds, rabbits, and, in one of his cerebellar experiments, a dog. These experiments led him to a small region of the brainstem that he called *le* *noeud de vie*, literally the “vital node” or “node of life.” Damage to it caused an abrupt termination of respiration and almost instantaneous death.

When ablating successive layers of the cerebellum, he found that his animals became unstable (at an intermediate point of extirpation) and would walk as if drunk. They stumbled around and had difficulty standing erect, yet appeared unaffected in other ways. These findings led Flourens to conclude, *contra* Gall, that the cerebellum coordinates skeletal muscular movements.

Turning to a still-higher level of the nervous system, Flourens conducted a series of experiments on the cerebral hemispheres. Using hens and pigeons, he ablated varying amounts of the hemispheres, sometimes with sequential lesions. Total or near-total ablations appeared to affect intelligence, judgment, and the ability to do things voluntarily (i.e., the will). He also observed a relative diminution of instinctive activities, while automatic or reflexive movements remained intact. Smaller ablations produced correspondingly smaller losses, and these were more likely to be temporary.

Flourens was still thinking in terms of the old, general faculties of perception, understanding, and volition/will, though now with a focus on the cerebrum. What he believed he was witnessing was that the degree of the disorder depended on the quantity of cerebral tissue removed, not whether the damage to the highest part of the brain involved specific territories in the front, top, back, etc. Moreover, his findings suggested that when one of these broad cerebral functions was affected, all were affected. These findings suggested cortical homogeneity.

Thus, Flourens was recognizing three basic nervous system functions at this time: sensibility, which he linked to perception and the will; excitability, which accounted for muscle contractions and movements; and movement coordination. Based on his results, he was now able to associate these three functions with different structures in the nervous system. As he saw it, the peripheral nerves directly excite the muscles; the spinal cord connects the peripheral nerves to the brain; the cerebellum coordinates movements in terms of regularity, course, timing, speed, and grip; and the cerebrum is responsible for willing, perception, and intellect.

Gall responded vigorously to Cuvier’s (1824) *Rapport*, which summarized Flourens’s experiments and conclusions for the *Académie*, since the young physiologist was not yet a member of the organization. He did so in the sixth (last) volume of his *Sur les Fonctions du Cerveau…*, in which he commented on newer publications. His scathing retort then appeared in the English translation of these volumes and in Combe and Combe’s (1838) collection of essays and other writings on the cerebellum.

Gall agreed with Flourens that the methods Haller and his followers had employed were poor. But he then argued that the same criticisms could be leveled against Flourens’s ablation experiments. It should be emphasized that Gall had always had his doubts about what could be learned by studying the effects of brain lesions, whether with humans or mutilated animals. How, he would ask rhetorically, could we be certain that the effects of ablation are limited to just the extirpated area? And “how can we remove one part, without involving the neighboring portions” (Gall, 1835, vol. 6, pp. 139–184)? He would answer that one simply cannot infer that, after damaging a specific part of the nervous system and observing the loss of some function, that ablated part is solely responsible for the lost function! And he would contend that, in order to draw conclusions about localization of function, “it is [first] necessary to know the special functions” (Gall, 1838b, p. 98).

“Suppose that M. Flourens wished to verify, by the ablation of the cerebellum, the point, whether this part be, or be not, the organ of the instinct of generation,” he further argued, “how will he make the animal live long enough to enable him to tell that it either possesses or has lost this instinct?” Additionally, “we should never lose sight of the fact, that the same part may possess its own general vital function, and its particular animal function” (Gall, 1838b, pp. 98–99).

Flourens’s choice of animals was another thing that, as put in Hallerian terms, clearly “irritated” him. How could anyone even begin to equate the brains of pigeons and hens, or even rabbits, with the infinitely larger and far more complex human brain, which was the primary subject of his own research program?

Gall concluded that “at most it is possible to obtain by these methods only some results, almost always very doubtful, in regard to the phenomena of irritation, of sensibility, of locomotive movements, and of the functions of certain viscera.” But, he continued, with such methods, “we shall never procure the least information concerning the special functions of the cerebellum, or of the particular parts of the brain (meaning cerebrum)” (Gall, 1838b, p. 100).

Gall was an animal lover, and he abhorred subjecting animals to painful mutilation experiments. Nonetheless, he now asked Spurzheim to perform similar experiments in his presence on hens, pigeons, and rabbits. These animals still showed themselves capable of perception and willed movements after removal of much of both hemispheres of the brain, with the rabbits even running and eating without help. He also conducted some brain lesion experiments with Giovanni Fossati (1786–1874) and others that led him to question all of Flourens’s ablation study findings.

That these attempts at replication provided different results from the Frenchman’s findings did not come as a surprise to Gall since he had recognized that even skillful surgeons are unable to replicate lesions adequately. In his words, “it is not possible to perform exactly the same operation, two or three times in succession, and to obtain always the same results” (Gall, 1838b, p. 106). But of greater importance was his repeated contention that ablation experiments “do not in the least enable us to decide whether a portion of the brain, and what portion of this organ, is indispensable to the execution of the functions of the senses with consciousness” (Gall, 1838b, p. 103). Consequently, and now with regard to Flourens’s findings: “Thus, every thing combines to prove that the notion that the cerebellum is the balancer and regulator of locomotive movements, is much more a singular idea than a true discovery” (Gall, 1838b, p. 113).

It is worth noting that Gall was not the only one who criticized Flourens’s work. Esteemed researchers François Magendie (1783–1855) in France, Silas Weir Mitchell (1829–1914) in the United States, and many others also criticized his experiments and conclusions (see Lechtenberg, 1994).

As with so many other things, Gall was absolutely certain he was right and his critics were mistaken. In the case of the cerebellum, however, time would show that Flourens was right and Gall was wrong, even though Flourens was not fully appreciating how difficult it is to draw firm conclusions about function–structure relationships by studying the effects of brain lesions on behavior or the problems inherent in generalizing from laboratory animals (especially birds and newborn animals, such as rabbits) to humans.

## Conclusion

Gall’s views about the cerebellum housing the organ for the reproductive instinct were formulated early on, during the mid-1790s, well before he left Vienna on his lecture tour through many German states and neighboring regions in 1805. Although reproductive instinct was not his most primitive faculty when he began, it emerged as his first faculty of mind before he entered Paris in 1807, where he promoted his new science for the rest of his life.

What changed over time was not just that Gall eliminated some faculties and their associated organs as he continued to work on his skull-based doctrine, but the amount and kinds of evidence he was able to amass for associating even his most primitive faculty of mind with the cerebellum. Guided by his craniology in humans and animals, but also drawing on cases of brain diseases and injuries, and even instances of human and animal castration, he dug in and fought to the end to defend the reproductive drive or instinct, which he tied so firmly to the cerebellum, as the first of his 27 faculties of the mind.

Without question, Gall was blinded by his faith in cranioscopy. And he was entirely too quick to dismiss clinical cases and other kinds of evidence challenging the conclusions he had formed about all of his organs of mind and their locations. This, of course, included the reproductive instinct and its organ in the cerebellum. In this broader context, what he wrote about the cerebellum and how he responded to Flourens can be regarded as representative of how he went about constructing and defending his doctrine.

In conclusion, we believe that historians have not looked carefully enough at Gall, his ideas, his methods, and his impact on nineteenth-century science and medicine. Gall was a naturalist of the mind with brilliant insights but also massive flaws—a man whose novel ideas and various methods are too often inaccurately and inadequately portrayed. Hopefully, this examination of the first of his final 27 faculties will serve as a stimulus for re-examining the thinking and contributions of one of the most important figures in the history of the neurosciences.

## Author Contributions

Both authors listed have made substantial, direct, and intellectual contribution to the work and approved it for publication.

## Conflict of Interest Statement

The authors declare that the research was conducted in the absence of any commercial or financial relationships that could be construed as a potential conflict of interest.
